# The Use of Industry 4.0 and 5.0 Technologies in the Transformation of Food Services: An Integrative Review

**DOI:** 10.3390/foods14244320

**Published:** 2025-12-15

**Authors:** Regiana Cantarelli da Silva, Lívia Bacharini Lima, Emanuele Batistela dos Santos, Rita de Cássia Akutsu

**Affiliations:** 1 Department of Nutrition, College of Health Sciences, University of Brasilia, Brasilia 70910-900, Brazil; liviabacharini@yahoo.com.br (L.B.L.); rita.akutsu@gmail.com (R.d.C.A.); 2 Department of Food and Nutrition, Faculty of Nutrition, Federal University of Mato Grosso, Cuiabá 78060-900, Brazil; emanuelebatistela.ufmt@gmail.com

**Keywords:** smart meal production, technology, automation, intelligent machines collaboration

## Abstract

Industry 5.0 involves the integration of advanced technologies, collaboration between humans and intelligent machines, resilience and sustainability, all of which are essential for the advancement of the food services industry. This analysis reviews the scientific literature on Industries 4.0 and 5.0 technologies, whether experimental or implemented, focused on producing large meals in food service. The review has been conducted through a systematic search, covering aspects from consumer ordering and the cooking process to distribution while considering management, quality control, and sustainability. A total of thirty-one articles, published between 2006 and 2025, were selected, with the majority focusing on Industry 5.0 (71%) and a significant proportion on testing phases (77.4%). In the context of Food Service Perspectives, the emphasis has been placed on customer service (32.3%), highlighting the use of Artificial Intelligence (AI)-powered robots for serving customers and AI for service personalization. Sustainability has also received attention (29%), focusing on AI and machine learning (ML) applications aimed at waste reduction. In management (22.6%), AI has been applied to optimize production schedules, enhance menu engineering, and improve overall management. Big Data (BD) and ML were utilized for sales analysis, while Blockchain technology was employed for traceability. Cooking innovations (9.7%) centered on automation, particularly the use of collaborative robots (cobots). For Quality Control (6.4%), AI, along with the Internet of Things (IoT) and Cloud Computing, has been used to monitor the physical aspects of food. The study underscores the importance of strategic investments in technology to optimize processes and resources, personalize services, and ensure food quality, thereby promoting balance and sustainability.

## 1. Introduction

A substantial proportion of the food service market comprises companies that still rely on manual labor and traditional equipment, such as pots, stoves, conventional ovens, and spreadsheet controls, particularly in developing countries [1]. This reflects a delay in adopting technologies that underscore the modern significance of food production and preservation. Moreover, the integration of technology within the food service sector is hindered by limited scientific research [2]. However, market diversification and the pursuit of technological advances, management, convenience, practicality, and new gastronomic experiences have driven the expansion and enhancement of services and operations in the food sector [3], especially in a world increasingly concerned with global sustainability.

Social and technological advances are becoming increasingly rapid and dynamic, significantly altering the service and production of food and meals, leading to substantial changes for the environment, the economy, and human health [4]. Current advancements support practices based on analysis and personalization, consumer profile monitoring, and agility. In this context, studying the technologies employed presents a valuable opportunity for technical, scientific, and practical improvements in the food service sector [4]. These advancements can generate new business opportunities, minimize food waste, reduce labor hours in meal production, and yield economic gains for businesses.

From the Industrial Revolution in the mid-1700s to the emergence of Industry 4.0, which began in 2011 and emphasized automation and digitalization, there has been a remarkable transformation in production processes [5,6]. Industry 4.0 focuses on smart production with objectives similar to those of previous revolutions: increasing mass productivity through innovative technologies of the time [7]. This includes the use of Artificial Intelligence (AI), Cloud Computing, and the Internet of Things (IoT) to enable real-time interfaces between the physical and virtual worlds [8,9,10].

The pace of adopting new technologies is accelerating rapidly, leading to Industry 5.0, which was defined in 2017 as a continuation of Industry 4.0. This phase proposes a sociotechnical evolution that aims to balance technological efficiency with a human-centered focus on the needs of current and future workers, human–machine interaction, and the personalization of processes and products. It also seeks to optimize energy consumption, materials processing, and product life cycle sustainably [4,11,12,13].

Industry 5.0 builds upon Industry 4.0 technologies and introduces several new innovations, including Edge Computing, Digital Twins, the Internet of Everything (IoE), Big Data analytics, collaborative robots (cobots), 6G, and Blockchain [9,14]. The key distinctions of Industry 5.0 relate to broader sustainability issues, such as the circular economy, cybersecurity, resilience in overcoming challenges, personalization, and a human-centered approach [15,16,17].

Within this framework, AI has produced significant advancements by analyzing consumption patterns, allowing for trend anticipation, personalization, and agile product development [16]. Evidence indicates that AI and other Industry 5.0 information and communication technologies can support efforts to quantify food production, enhance traceability and food safety, reduce waste, and improve efficiency [17,18].

Food services operating within the Industry 5.0 model can significantly enhance production methods when properly structured [4]. Understanding the technologies and concepts being proposed and utilized globally in Industries 4.0 and 5.0 can highlight unique opportunities for the development of the food service sector. This understanding can also expedite awareness of these new realities, as well as facilitate discussions on potential barriers to adopting these technologies.

Therefore, this integrative review aims to analyze the scientific literature on Industry 4.0 and Industry 5.0 technologies in the food service sector. The goal is to provide insights into global trends and challenges related to the implementation of these technologies, with particular emphasis on sustainability, efficiency, and personalization. The novelty of this work lies in classifying technologies by functional perspectives and highlighting the geographical distribution of the research.

## 2. Materials and Methods

This integrative review has been conducted using a systematic search that follows the recommendations set out by the Preferred Reporting Items for Systematic Reviews and Meta-Analyses (PRISMA) [19] in order to enhance the quality of the review process. We completed the PRISMA checklist (see Appendix A) and registered the review with the Open Science Framework (OSF), accessible at https://osf.io/yg27t (accessed on 10 October 2025).

An integrative review has been selected, as it allows for the inclusion of studies with various methodologies, thereby providing a broader and more comprehensive analysis of the topic [20]. This area of research is relatively new, with limited publications in the food production field.

The research question guiding this review has been: “What Industry 4.0 and 5.0 technologies are available for application in food services?”

### 2.1. Eligibility Criteria

This study has considered original articles that have addressed the use of Industry 4.0 and 5.0 technologies in food service operations producing large meals (lunch and/or dinner), specifically in the stages directly associated with the production process (customer order, preparation, cooking, and distribution). The inclusion of Industry 4.0 studies is justified for the following reasons: (I) It is part of the evolutionary path leading to Industry 5.0 technologies and processes; (II) the adoption of concepts and practices from these two industries occurs at different times and levels within each country; and (III) it is challenging to distinctly separate developments that have occurred between 2011 and 2017.

It has considered technologies that have been tested, are under testing, or have been implemented. Publications are not restricted by either language or publication date.

The following exclusion criteria were applied: (I) reviews, letters, books, conference or congress articles and abstracts, discussions, reviews, and undergraduate final papers; (II) papers focused on deliveries, payment methods, and improvements for robotic systems; (III) interviews about innovations; (IV) production of coffee shop meals, small meals, and snacks; (V) assessments of customer satisfaction; (VI) restaurant selection systems; (VII) modifications limited to the physical structure of food services; and (VIII) technologies related to other historical eras of the industry.

### 2.2. Information Source

Detailed individual search strategies have been developed for the following databases: Medline (PubMed), Lilacs, Web of Science, Scopus, and Embase. A search for gray literature has been conducted using Google Scholar. Additionally, the reference lists of selected articles have been reviewed to identify relevant studies that may have been overlooked during the electronic searches in the databases. Initial searches across all databases were conducted in March 2024, with an update on 17 March to include the most recent articles.

### 2.3. Search Strategy

Appropriate keywords have been selected using the Medical Subject Headings (MeSH) database of the National Library of Medicine, along with insights gained from reading articles in the field. These keywords have then been adapted for searches within each database. Descriptors and their combinations have been used in both Portuguese and English to search for articles.

The descriptors have been divided into two groups.

#### 2.3.1. Group 1

Group 1 consists of terms that characterize food services and their synonyms; singular and plural terms (“Food Service”, “Catering service”, “Meals on Wheels”, “Restaurant”, “Fast Food”, “Convenience Food”, “Ready-Prepared Food”, “Ready-To-Eat Meal”, “Restaurante”, “Serviço de alimentação”).

#### 2.3.2. Group 2

Group 2 consists of terms correlated with Industries 4.0 and 5.0 and their synonyms; singular and plural terms (“Industry 4.0”, “Industry 5.0”, “Indústria 4.0”, “Indústria 5.0”, “Automation”, “Automatic”, “Meal delivery system”, “Robot applications”, “Information and communication technologies”, “IoT”, “Robots”, “Cloud computing” “Cloud Processing”, “Cloud Storage”, “Cloud Service”, “Edge Computing”, “Fog Computing”, “Artificial intelligence”, “Computational Intelligence”, “Machine Intelligence”, “Computer Reasoning”, “AI”, “Computer Vision System”, “Knowledge Acquisition”, “Knowledge Representation”, “Machine learning”, “Transfer Learning”, “Big data analytics”, “Digital twin”, “Blockchain”, “Block Chain”, “Internet of Everything”, “Cobots”, “Autonomous navigation”, “Emerging Technologies”).

The Boolean operator “OR” has been used between the terms of each group, and the Boolean operator “AND” has been used between Group 1 and Group 2. Rayyan software (https://www.rayyan.ai/) (Qatar Computing Research Institute—QCRI) has been used to assist in selecting and excluding duplicate articles.

### 2.4. Study Selection

The screening process for studies has been conducted in two phases. In Phase 1, two researchers independently reviewed the titles and abstracts of all references identified in the databases, excluding articles that did not meet the eligibility criteria. In Phase 2, the same reviewers read the full texts of the selected articles, including only those that met the inclusion criteria.

If there was a disagreement between the two reviewers, they held discussions until a consensus was reached for both phases. If they could not come to an agreement, a third reviewer made the final decision. In cases of lingering uncertainty, an expert provided the final judgment. The final selection was based on the full text of the articles, and one reviewer compiled a critically evaluated reference list of the selected studies.

### 2.5. Data Collection Process

Two reviewers independently gathered the following characteristics from the selected studies: authors and year of publication, country of research, study objectives, the specific technologies aligned with Industry 4.0 or 5.0 that were addressed, the Perspective of the food service relevant to the technology, the type of sample used, whether the technology was in the testing phase or had been implemented, and the main results and limitations of each study.

The data extraction form has been developed by the review team and has undergone testing with a pilot group of five articles for refinement and team calibration. Data analysis has been conducted using descriptive statistics, which have been presented as absolute and relative frequencies.

**Figure 1:**
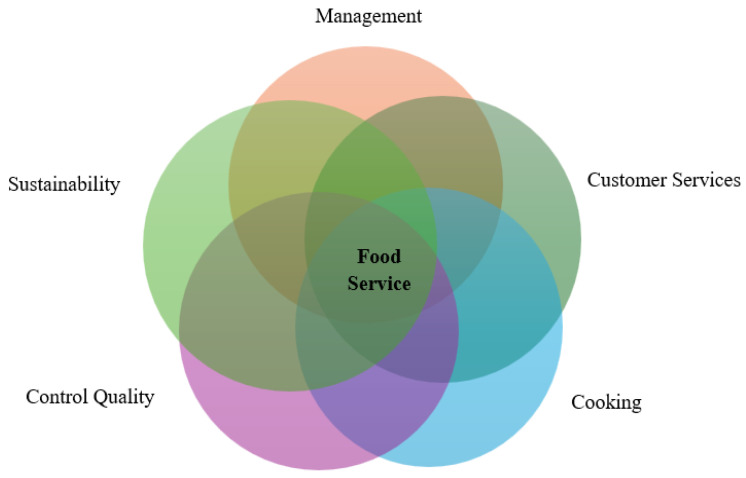
Food Service Perspectives. Source: elaboration of the authors, 2025.

If the country of data collection has not been specified in a study, it has been attributed to the country where the main author had listed their address or affiliation. To identify the type of technology, the concepts of Industry 4.0 and 5.0 have been analyzed to determine their respective eras. The studies have been classified according to the technologies adopted within different service perspectives, including customer service, cooking, management, sustainability and quality control, as illustrated in Figure 1.

The naming of these perspectives is original to the reviewers and was developed based on their practical experience. The aim was to create a more direct and practical classification while considering the themes addressed in the articles and study materials. Classification was based on the primary objective of each study, with some studies presenting more than one Perspective.

The Customer Service Perspective involves technologies used for receiving customers, taking orders, providing direct and in-person service, and clearing tables. The Sustainability Perspective focuses on technologies that help identify and reduce waste production, as well as conserve natural resources. The Management Perspective includes technologies that assist in forecasting customer demand, planning, and analyzing administrative and operational processes in food service. The Cooking Perspective pertains to the technologies used for the direct preparation and production of food. Quality control involves technologies that promote microbiological safety, preservation, and the organoleptic quality of food.

In analyzing these Perspectives, we have considered technologies that have been tested or are still in prototype stages within food service, all related to direct and in-person customer service.

## 3. Results

### 3.1. Selection of Studies

**Figure 2:**
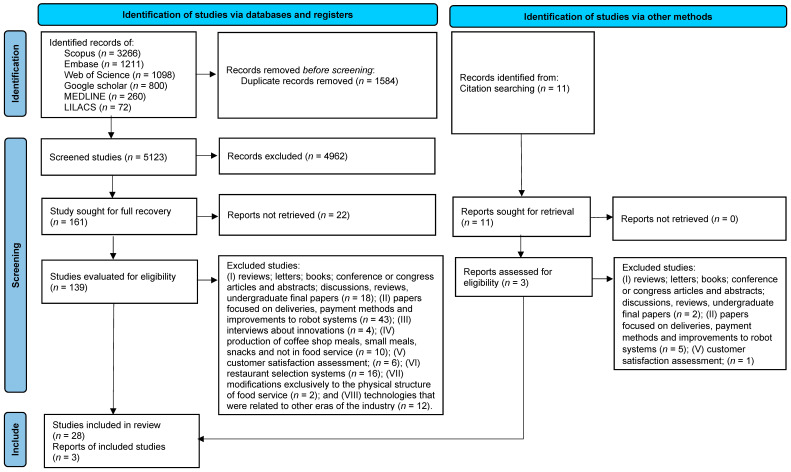
Flowchart of the integrative review search process. Adapted from the PRISMA protocol. Source: Page et al. [19].

A total of 6707 studies were identified in the databases, and 1584 duplicates were excluded. Thus, of these, 4962 have been excluded for not having met the eligibility criteria, and 161 have been selected for full-text review. However, it has not been possible to access 22 studies for full reading due to difficulties in contacting the authors (for example, due to their passing) or the magazines (such as the discontinuation of older print journals). This has resulted in 139 studies being considered eligible (see Figure 2).

After a full-text review, 28 studies have been deemed suitable for inclusion. Among these, 24 studies (77.4% of the total selected) have been obtained from the initial database search conducted in March 2024, while four articles (12.9% of the total selected) have been obtained from the last search in March 2025. Additionally, 3 studies identified in the references of the selected articles have also met the eligibility criteria, bringing the total to 31 selected studies (Figure 2).

### 3.2. Studies’ Characteristics

Among the selected studies, the majority have been conducted in Asian countries, accounting for 54.8% (*n* = 17). In Europe, 11 studies have been identified, representing 35.5%, while the Americas have contributed 3 studies, or 9.7%. No articles have been selected from Africa or Oceania.

**Figure 3:**
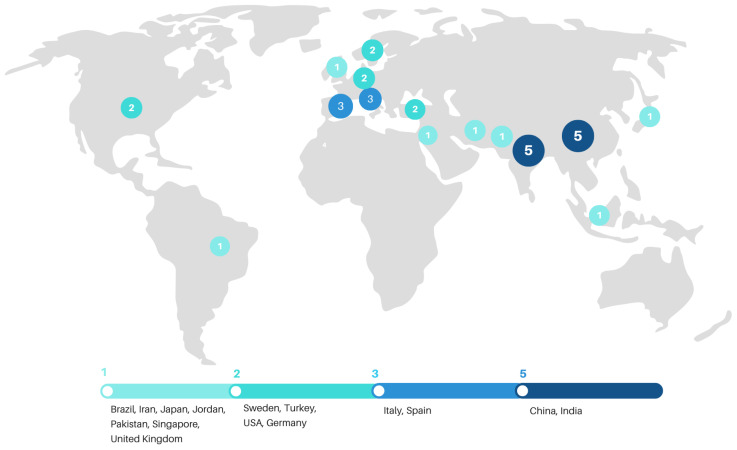
Distribution of selected articles by country. Source: study data.

The studies have been conducted in the following countries: China (16.1%, *n* = 5), India (16.1%, *n* = 5), Italy (9.7%, *n* = 3), and Spain (9.7%, *n* = 3). Germany, the United States, Sweden, and Turkey each contributed 6.5% (*n* = 2 each). Brazil, Iran, Japan, Jordan, Pakistan, Singapore, and the United Kingdom each accounted for 3.2% (*n* = 1 each) (see Figure 3).

Table 1 outlines the key characteristics of the selected articles, detailing the service Perspective, author(s), year of publication, a summary of the technologies discussed, and the objectives of the studies. Additional information, such as the type of industrial evolution with which each study aligns, whether Industry 4.0 or 5.0, can be found in Appendix B.

Among the selected studies, 77.4% (*n* = 24) are currently in the testing phase. Furthermore, the majority of these studies have been published between 2011 and 2017, which corresponds with the emergence and development of Industries 4.0 and 5.0 (as shown in Table 1 and Appendix B).

**Figure 4:**
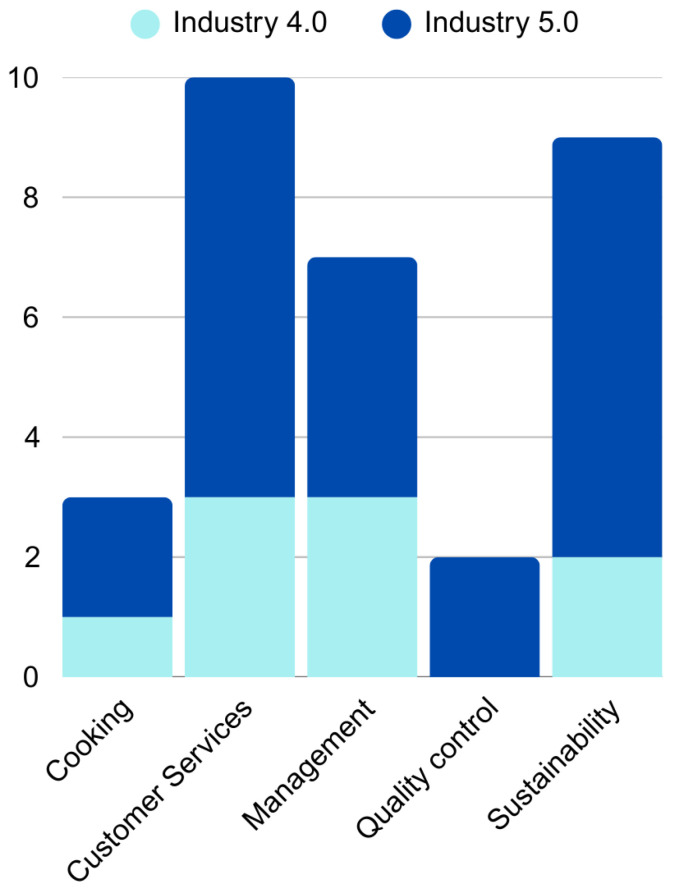
Distribution of service Perspectives among selected studies. Source: study data.

Most of the selected studies (71%, *n* = 22) refer to Industry 5.0 technologies (Figure 4) and have been tested or implemented in commercial restaurants.

**Figure 5:**
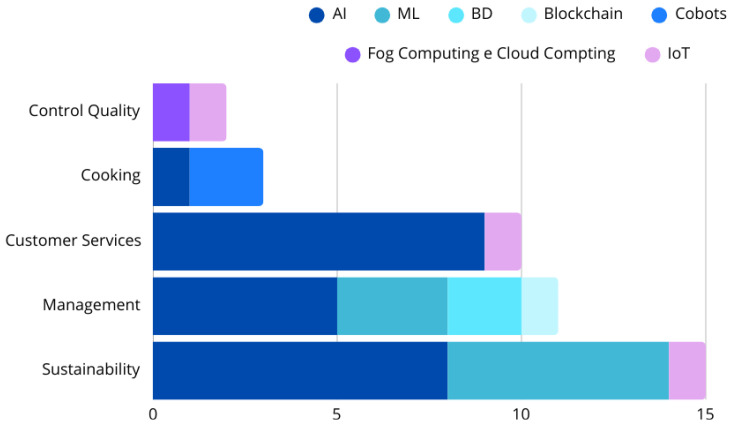
Distribution of the types of technologies reported in the studies. Source: study data.

In the realm of Food Service Perspectives (Figure 1 and Figure 4), customer service has emerged as the most frequently addressed theme, comprising 32.3% of the total studies (*n* = 10), with 70% (*n* = 7) of these focusing on elements of Industry 5.0. Within this category, research has highlighted the use of AI-powered robots for customer service (*n* = 5), menu recommendation, and service personalization for patrons (*n* = 4). Additionally, the utilization of technologies such as IoT and QR codes has facilitated self-service and enabled the real-time traceability of orders and inventory items (*n* = 1) [24,25,27,28,29,30,31,32,33] (Figure 5).

The researchers have noted that despite advancements in mobile service robots driven by AI, most still require improvements in mobility, agility, and communication, with optimistic expectations for enhanced functionality.

In the Cooking Perspective, which accounts for 9.7% (*n* = 3) of the total selected studies, articles have discussed the automated identification of grain cooking points (*n* = 1) and cooking automation using cobots (*n* = 2) [21,22,23] (Figure 5).

The Management Perspective represents 22.6% (*n* = 7) of the total. Studies in this category report the use of computational systems with AI to schedule production agendas (*n* = 1), support menu engineering (*n* = 1), and assist with general restaurant management (*n* = 2) [34,35,36,37]. Additionally, BD and ML have been employed to analyze sales (*n* = 2), while Blockchain (*n* = 1) is associated with traceability and the comprehensive analysis of logistics data [38,39,40] (Figure 5). Technologies for demand prediction, which also influence management, have been primarily utilized to promote sustainability.

Quality control, representing 6.4% (*n* = 2), has involved studies that referred to the application of AI, IoT, Fog Computing, and Cloud Computing to evaluate food quality in real time, monitoring essential physical aspects such as temperature and humidity [41,42] (Figure 5).

The Sustainability Perspective accounts for 29% of the total (*n* = 9), with the most studied pillar being waste reduction. Research in this area has largely focused on leveraging AI and ML to predict meal demand and improve food production planning, ultimately reducing food waste and achieving economic benefits [44,47,48,49,51]. One study provided a broader view of the environmental impacts associated with food preparation and consumption [48], while another discussed resilience during crises such as the COVID-19 pandemic [44].

AI and ML have also been utilized to monitor gas emissions and visual indicators of refrigerated food spoilage [46]. Additionally, AI combined with computer vision have been used for the identification of served and wasted food [49]. Two studies employed IoT technology: one aimed at detecting anomalies and discrepancies in food waste disposal to enhance food production scheduling [43], while the other collected data on meal reservations and attendance rates, enabling better meal production adjustments and cost reduction [45] (Figure 5).

Regarding the types of technology employed, AI was the most frequently utilized element, both on its own and in combination with other technologies, followed by ML and IoT. Cobots, BD, and Blockchain were referenced less often (Figure 5).

Figure 5 illustrates the number of studies that employed each technology, whether individually or concurrently.

**Figure 6:**
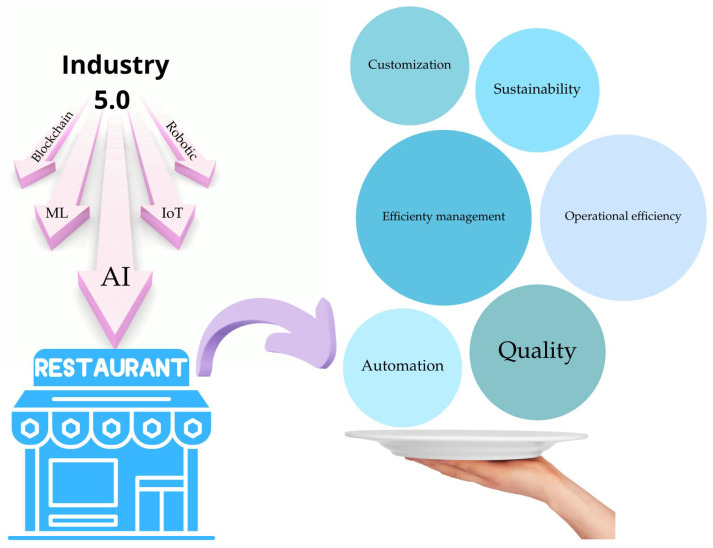
Illustrates Industry 5.0 and its benefits for the food service sector. Source: elaboration of the authors, 2025.

Figure 6 succinctly illustrates the technological convergence of Industry 5.0 and its resulting benefits for the food service sector.

## 4. Discussion

The results of this integrative review confirm that Industry 4.0 and 5.0 technologies in the food service sector have the potential to improve the quality, speed, and personalization of customer service. Additionally, this study highlights that these technologies can enhance processes, facilitate human–machine interaction, and optimize meal production planning, all while reducing environmental impacts and improving the financial performance of the sector.

The predominance of selected studies in the testing phase underscores the emerging and innovative nature of Industry 4.0 and 5.0 technologies within the food service industry. This also emphasizes the need for further research to assess the feasibility of these technologies in real-world settings. Most importantly, it is crucial to investigate how these technologies can help tailor work to the conditions of the workers, such as their height and physical strength, as well as reduce occupational illnesses, especially since these environments are often very demanding.

This expansion of research in the area can contribute to overcoming the challenges associated with the incorporation of innovative technologies: scalability, high initial costs, the need for restructuring the production environment and professional training, as well as promoting technological acceptance, among others [13].

Considering these challenges, it is projected that by 2030, 59% of the global workforce will require reskilling due to the adoption of new technologies [52]. This reality necessitates strategic investments for the training and reskilling of workers, focusing on critical thinking and flexibility skills that will be complementary to the automation of monotonous and risky tasks (such as some activities in the cooking process). Furthermore, investments are needed in infrastructures and public policies that promote these conditions.

### 4.1. Distribution of Studies by Country or Region

An example of these strategic investments mentioned above is the leveraging of Industry 4.0, which formally began through the German government program “Industrie 4.0”; this made it possible to increase the country’s economic competitiveness in the industrial sector [17]. In this regard, other countries also made similar investments [17]. Likewise, for advances toward Industry 5.0, countries such as the pioneer Japan (with the “Society 5.0” program) and subsequently the European Union, China, India, and the USA launched public policies for the construction of a skilled workforce, digitalization, and the development of industry and the economy [11,17,53]. Likely related to these political incentives, this review observes a predominance of studies concentrated in these regions.

It is observed that developed countries maintain their historical importance in the development of innovative technologies and the adoption of advanced industrial practices [13].

Incentive programs targeting the food service sector, modeled after global standards, seem necessary, particularly in developing countries, to enhance formality, economic competitiveness, social equity, and sustainability [52].

The limited dissemination of knowledge and education across all hierarchical levels, from management to operations, coupled with restricted infrastructure and financial resources, likely hinders the implementation of Industry 4.0 and 5.0 in developing countries [8,14,16,54], thus justifying the low representation of Africa, Central America, and South America in this review.

Considering this data, it is evident that, in 2024, Brazil exported the world’s largest volume of processed foods, and its major industry is food and beverages (accounting for 10.8% of its Gross Domestic Product (GDP)) [55]. This is largely due to the extensive incorporation of technology by the private food sector, particularly in agriculture [55]. Despite the acknowledged need for greater public infrastructure, this serves as an example of how developing nations can achieve prominence through technological investment. This model can be extrapolated to the food service sector.

Another example of investments in development is China, an epicenter for the adoption of Industry 5.0-oriented technologies and one of the leading global innovators in technological advancements. China directs substantial investments toward large-scale automation (employing robots, cobots, IoT, and IoE), AI, and process integration in both the service and industrial sectors [54].

India, another developing country, has been standing out for the adoption of practical skills technologies in the service and hospitality sectors, prioritizing more adaptable and cost-effective technological solutions (such as digital menus accessible via QR codes and ordering and payment systems using digital wallets) [56].

Indian startups are also driving the development of advanced technologies such as AI, robotics, and IoT [53], observed in this review, to improve manufacturing processes, increase productivity, and reduce costs. Examples include the autonomous quality control of food storage and the robotization of customer service and management systems.

The absence of more food service studies in a wider range of countries, as discussed above, is likely also due to the preservation of competitive strategies, a focus on internal results, and the intrinsic nature of the service sector. This sector generally produces fewer scientific publications compared to industrial sectors such as automotive and manufacturing, but it has been increasingly investing in technology, and innovation [57].

### 4.2. Perspectives and Employed Technologies

Following the Perspectives distribution adopted in this study, the results reveal a strong emphasis on sustainability, which goes hand in hand with innovation. Sustainability is defined as the integration of actions focused on environmental, social, and economic pillars, with the aim of seeking quality of life and environmental balance for a better standard of development [58].

Within this Perspective, the application of AI, ML, and IoT demonstrates the potential to promote more sustainable practices and efficiency in the sector, such as predicting meal demand, optimizing inventory, identifying signs of food spoilage (or food deterioration), and better detecting critical points of waste generation.

The utilization of sensors integrated into technological systems allows for more precise monitoring of the type and volume of discarded waste, assisting in production management and in actions to minimize the environmental impacts of its generation [51]. Furthermore, obtaining a more accurate understanding of production and waste data can also lead to team training and involvement, stimulating creativity and further advancements in menu engineering, such as for whole food utilization and the reuse of leftovers in differentiated recipes [36].

It is important to note that these technologies, besides influencing production costs, also impact the social sphere, since they reduce the workload of employees, prevent the production of food volumes that will be discarded, and minimize the environmental impacts caused by meal production [48].

However, technologies utilizing renewable energy and the transformation of waste into other products (such as organic waste into fertilizer and biofuel) were not present for the evaluation of the Sustainability Perspective. It would be relevant to assess these points in food services given the known impact of energy consumption and the type of energy utilized, as well as the waste generated by the sector and its environmental challenges (or problematic impact on the environment) [13].

Among the identified perspectives, customer service stands out by revealing the potential of Industry 5.0 to enhance the consumer experience by integrating human–machine interaction, service personalization, and customer loyalty strategies [4].

This has been corroborated by research in Brazil, which indicates that food service customers seek tastier, healthier, hygienically prepared, high-quality, and accessible food and that the service should be friendly, personalized, convenient, and adopt digital technologies for autonomy [59]. This is, in part, aligned with the proposals of Industry 5.0.

The incorporation of AI-enabled robots for service and delivery and the provision of recommendations based on consumption history/preferences using AI and self-service features via IoT demonstrate a trend toward the digitalization of services. These technological solutions contribute not only to operational efficiency but also to the creation of more personalized, autonomous, and convenient experiences, aspects highly valued by consumers [60].

Concerning the use of robots in the workplace, the International Federation of Robotics (IFR) World Robotics Report has revealed a 70% increase in the deployment of new robots overall, with the hospitality sector being the second-largest employer of robots in the global service sector [61]. Besides robots, the strongest global trends in the sector are the adoption of AI, ML, and Digital Twins [62].

Nevertheless, autonomous waiter-type service robots still face challenges in mobility (difficulties in overcoming objects and on some surfaces) and efficient human interaction (such as language failures and interpretation of human speech), in addition to high acquisition and maintenance costs, although these are expected to improve with technological advancements [4]. These critical points favor human service in dine-in restaurants, which is also reinforced by the greater human capacity for flexibility and adaptability in service [12].

In addition to the incorporation of robots, other technologies have contributed to the transformation of the customer experience in food service, especially by increasing autonomy, expediting processes, and personalizing service.

In this context, modern cloud-based point-of-sale (POS) systems stand out, which, despite having lower initial costs, are scalable and feature modular functionalities, such as online order management and loyalty programs [12]. Online digital scheduling and reservation tools optimize table occupancy and reduce the amount of time spent on telephone service, with many being accessible by small restaurants [59].

With the COVID-19 pandemic, the practice of scanning customers using QR codes with their smartphones to view digital menus became common [59]. This simple technological employment greatly benefits the food service sector, as it reduces printing costs, facilitates real-time updates of dishes and prices, and can offer language options, information about ingredients, allergens, and photos of the dishes.

Additionally, the use of mobile applications or simple web platforms, through which customers may place their orders directly, expedites the ordering process and reduces the potential for communication errors between customers and employees [33,53]. These technologies, aligned with the principles of Industry 5.0, reinforce consumer centrality, promoting integration between autonomy, convenience, and technological humanization in customer service.

Management is one of the fundamental pillars of Industry 5.0 due to the necessity of integrating smart systems, sustainable practices, and data-driven decisions [16,17]. Consequently, studies within this Perspective highlight the use of AI, BD, ML, and Blockchain in order to optimize internal processes such as production scheduling, menu engineering, procurement planning, storage, sales analysis, and logistical traceability [38].

More efficient management generates greater process fluidity and higher overall quality, which can be perceived by customers and by the employees themselves, in addition to generating higher profits, thereby reinforcing the relevance of technological adoption in this Perspective.

In the Cooking Perspective, the focus is on automation through cobots and the identification of cooking points. These technologies are particularly relevant, because they allow for greater standardization and efficiency in meal production, reducing human errors, inaccuracies, and optimizing resources, thereby establishing a standard of quality and identity [52,62]. Consequently, they can infer greater sustainability in the production chain (such as less waste generation and reduced use of resources—materials, energy, and water).

Nevertheless, it is necessary to evaluate cobots in food production regarding the safety of their components in the kitchen environment and the potential risks of cobots in the handling of knives, chemicals, and intense heat when working collaboratively with humans [4].

Thus, there is potential for greater exploitation of technological employment in cooking given the possibility that tasks involving greater physical risk and monotony be assigned to robots and machines, reserving tasks that require critical thinking for humans [14].

Advanced cooking equipment, such as multifunctional cooking centers and combi-ovens with AI and IoT, allows for higher sensory quality of food, remote programming, optimization of the preparation process, and increased productivity with fewer pieces of equipment, contributing to resource savings, improved thermal comfort, and better utilization of physical space [3].

The characteristics of this equipment also have the potential to favor sustainability by providing greater energy efficiency, improved preparation yield, and waste reduction, provided they are adequately parameterized and operated [3].

Despite its potential, this technology has not been contemplated among the findings of this review, indicating a gap in relation to the investigation of advanced cooking solutions within the context of Industry 5.0 applied to food services.

In the same vein, no studies on 3D food printing have been identified. Three-dimensional printing has made it possible to provide personalized nutrition and is conducive to the reduction in food waste [62]. Furthermore, it can help chefs have a less physically demanding job, allowing them to concentrate on the gastronomic excellence of crucial preparation stages and the finishing of dishes.

The last Perspective analyzed in this study is the quality control approach, which stands out in the use of AI systems with IoT, Fog Computing, and Cloud Computing. These have been employed for the real-time monitoring of food preservation characteristics (such as color changes and gas emissions), demonstrating the capacity to ensure higher standards of safety and quality. Monitoring these aspects can also reduce the discarding of food.

Another form of quality control is the interconnection between sensors and smart systems, employing AI and Blockchain, which significantly enhances production chain traceability, increasing food safety, ensuring product authenticity, and strengthening consumer trust [17,63,64].

Technological employment to enhance quality control in food services, besides the aspects referred to above, favors the identification of failures and thus opportunities for improvement. It also contributes to reducing costs, increasing profitability, customer loyalty, and compliance with legislation [17].

Finally, the findings of this review reflect the core technologies of Industry 4.0 and 5.0 as well as their potential application in the food sector. The combination of these technologies enables the development of smart and automated systems that can optimize processes whilst valuing human capabilities, enhancing the customer experience, and promoting sustainability and the circular economy.

### 4.3. Study Limitations

This study presents several limitations that must be considered. Firstly, the work involves a diversity of study methodologies due to the theme being broad and unique (or singular), and which differs from the topics that are commonly researched in nutrition.

Linguistic barriers stemming from articles not written in English, which required additional effort for translation and reading, was also a limiting factor.

Finally, another limitation concerns the exclusion of studies focused on delivery services. Although these services are growing and represent a relevant field of technological innovation in the food sector in general, it has been considered that delivery constitutes a distinct stage more associated with the distribution and logistics of food services, whereas the present study has focused on the chain of production, preparation, and direct customer service within establishments. Thus, it is understood that the analysis of technologies applied specifically to delivery deserves to be explored in studies that are exclusively directed at this emergent phenomenon.

This integrative review has allowed for the identification and mapping of Industry 4.0 and 5.0 technologies available for the food service sector, with an emphasis on sustainability and customer service, highlighting a paradigm shift toward optimization, humanization, and reduced waste generation.

It has been observed that the adoption of technologies such as AI, IoT, ML, and robotics transcends operational efficiency, promoting sustainability with an emphasis on a reduction in food waste, and also, greater quality, speed, and personalization of customer service. These technologies strengthen Industry 5.0’s focus on sustainability and human–machine interaction to enhance the consumer experience and management.

Technological innovation in food service appears to be intrinsically linked to governmental policies that incentivize the advancement of infrastructures, knowledge, and worker training. This establishes that the advancement of the sector is favored by a robust public–private support ‘ecosystem’, which can also contribute to improving the geographical distribution of access to cutting-edge technologies.

The findings of this review have urgent practical implications for stakeholders within the food service sector, governments, and educational institutions. It is recommended that food service managers invest in low-cost and rapidly deployable technologies (such as digital QR menus and cloud-based self-service and point-of-sale systems), which have proven to be highly effective and accessible, widely impacting daily life.

It is also recommended that the adoption of tools such as AI and ML for demand forecasting be prioritized to optimize inventory and minimize costs and waste.

The identified gaps underscore the need to deepen research into technologies that are still underexplored in the sector, such as 3D food printing, multifunctional cooking equipment, and renewable energy sources, which can contribute to a more efficient and sustainable food service model. It is recommended that the evaluation of the implementation of Industry 4.0 and 5.0 technologies be included in real-world environments and diverse cultural and economic contexts (especially in developing countries), considering feasibility, return on investment (ROI), human impact, and the long-term impact of the implementation. Furthermore, it is recommended that the impacts of technologies on sector workers, with considerations of reskilling, remuneration, new roles (or functions), and well-being, be explored in greater depth.

It is concluded that the technologies of Industry 4.0 and 5.0 possess transformative potential for the food service sector, allowing for the integration of sustainability, innovation, smart automation, and human valuation. It is expected that this work will contribute to raising awareness among stakeholders in the food service sector about the possibilities of employing technologies in gastronomy, nutritional care, management, customer service, and sustainability.

## 5. Conclusions

The literature analysis indicates a growing interest in the application of Industry 4.0 and 5.0 technologies in the food service sector, with a significant emphasis on sustainability and customer service. Most studies are still in the experimental phase due to the recent and innovative nature of the topic, highlighting a dynamic and evolving field.

The geographical distribution of the studies reveals a global interest in the subject, as well as a need for further political incentives and investigation in regions such as Oceania, the Americas, and Africa.

AI and ML emerge as the most promising technologies for transforming the food service industry. Future research directions include the evaluation of practical implementation, economic implications, and the human impact of the adoption of these technologies.

This work aims to inspire nutritionists, chefs, administrators, and business owners in the food service sector to explore the potential of employing these technologies in gastronomy, nutritional care, management, and social and environmental contexts. It also encourages advancements in catering, food service establishments, and related fields.

## Figures and Tables

**Table 1:** Main descriptive characteristics and results from studies.

Service Perspective	Reference	Technology	Objective
Cooking	Anami, Burkpalli (2009) [21]	AI	Monitor cooking
	Junge et al. (2020) [22]	Cobot	Automation cooking
	Ronzoni et al. (2021) [23]	Cobot	Automation processes cooking
Customer Services	Acosta et al. (2006) [24]	Robot with AI	Collect utensils from tables
	Tan et al. (2011) [25]	AI	Identify customers and recommend personalized menus
	Cheong et al. (2016) [26]	Robot with AI	Assist and serve customers
	Li et al. (2018) [27]	AI	Predict consumer food preferences, recommendations, and service personalization
	Roanes-Lozano et al. (2019) [28]	AI	Personalize menus to the customer’s dietary characteristics and the availability of ingredients in the restaurant
	Tallam, Joseph (2019) [29]	Robot with AI	Assist and serve customers
	Chen et al. (2022) [30]	Robot with AI	Serve customers
	Huang et al. (2022) [31]	AI	Choose the ideal schedule for the production and delivery of dishes to customers
	Shimmura et al. (2023) [32]	Robot with AI	Serve customers
	Sultana et al. (2024) [33]	IoT and QR code	Fulfilling customer orders and monitoring the traceability of the production chain
Management	Blasi (2018) [34]	AI	Manage production schedule
	Sun et al. (2018) [35]	ML	Loss prevention
	Tufano et al. (2018) [36]	BD * and ML *	Sales analysis, inventory, and purchasing management
	Tom, Annaraud (2021) [37]	AI	Menu engineering support
	Gómez-Talal et al. (2024) [38]	BD and ML	Analyze customer sales
	Groene, Zakharov (2024) [39]	AI	Sales forecasting
	Hao et al. (2024) [40]	Blockchain	Food supply chain
Quality Control	Bhatia (2020) [41]	IoT	Food quality control
	Bhatia, Manocha (2022) [42]	AI, Fog Computing, and Cloud Computing	Evaluation of food quality in real time
Sustainability	Aytaç, Korçak (2021) [43]	AI, ML and IoT	Production quantity planning and monitoring food waste to reduce its generation
	Eriksson et al. (2021) [44]	AI and ML	Predict guest attendance to reduce the food waste generated
	Faezirad et al. (2021) [45]	IoT	Predict guest attendance to reduce the food waste generated
	Gull et al. (2021) [46]	AI and ML	Monitor food deterioration signals for quality control and reduce the waste generated
	alefors et al. (2021) [47]	AI and ML	Predict guest attendance to reduce the food waste generated
	Principato et al. (2023) [48]	AI	Reduce the food waste generated
	Rodrigues et al. (2024) [49]	AI and ML	Predict guest attendance to reduce the food waste generated
	Sigala et al. (2025) [50]	AI	Food waste analysis system to reduce the waste generated
	Turker (2025) [51]	AI and ML	Predict guest attendance to reduce the food waste generated

* Big Data (BD), machine learning (ML).

## Data Availability

The original contributions presented in this study are included in the article. Further inquiries can be directed to the corresponding author.

## Glossary

AI—Artificial Intelligence	A branch of science that allows machines to learn, reason, and act like humans [17].
BD—Big Data	A comprehensive and varied collection of data gathered from multiple sources, including consumption, customer, product data, and potential routes [17].
Big Data Analytics	The analysis of large and diverse datasets from various sources aims to define distinct profiles [12].
Blockchain	A decentralized management system for various connected heterogeneous devices, characterized by immutability, encryption, resistance to data tampering, and enhanced security. This system is applicable for traceability and monitoring of orders, payments, accounts, and other transactions [16].
Cloud Computing	A technology that enables remote access to information technology services [17].
Cobot—Collaborative Robotics	Enhances flexibility and automation in assembly lines, utilizing collaborative robots in restaurants for serving customers, delivering food, and collecting and washing dishes [17].
Deep Learning	A branch of AI used for complex pattern recognition tasks, such as image classification and object detection, because of its ability to learn hierarchical data representations [4].
Digital Twins	A virtual representation of a physical object or system [17].
Edge Computing	Enables data processing and the execution of actions in real-time [17].
IoE—Internet of Everything	Integrating devices, people, and processes [4].
IoT—Internet of Things	Solely considers the connection between devices [4].
ML—Machine Learning	A subset of AI that employs algorithms to analyze data and learn from it [4].
Sixth-generation wireless (6G)	Multi-layered, highly dynamic broadband [12].

## Appendix A

**Table A1:** PRISMA-ScR Checklist.

Section	Item	PRISMA-ScR Checklist Item	Reported on Page
**Title**			
Title	1	Identify the report as a scoping review.	1
**Absctract**			
Structured summary	2	Provide a structured summary that includes the following (as applicable): background, objectives, eligibility criteria, sources of evidence, charting methods, results, and conclusions that relate to the review questions and objectives.	1
**Introduction**			
Rationale	3	Describe the rationale for the review in the context of what is already known. Explain why the review questions/objectives lend themselves to a scoping review approach.	2–3
Objectives	4	Provide an explicit statement of the questions and objectives being addressed with reference to their key elements (e.g., population or participants, concepts, and context) or other relevant key elements used to conceptualize the review questions and/or objectives.	3
**Methods**			
Protocol and registration	5	Indicate whether a review protocol exists; state if and where it can be accessed (e.g., a web address); and, if available, provide registration information, including the registration number.	3
Eligibility criteria	6	Specify characteristics of the sources of evidence used as eligibility criteria (e.g., years considered, language, and publication status) and provide a rationale.	3–4
Information sources	7	Describe all information sources in the search (e.g., databases with dates of coverage and contact with authors to identify additional sources), as well as the date the most recent search was executed.	4
Search	8	Present the full electronic search strategy for at least 1 database, including any limits used, such that it could be repeated.	5
Selection of sources of evidence	9	State the process for selecting sources of evidence (i.e., screening and eligibility) included in the scoping review.	5
Data charting process	10	Describe the methods of charting data from the included sources of evidence (e.g., calibrated forms or forms that have been tested by the team before their use and whether data charting was performed independently or in duplicate) and any processes for obtaining and confirming data from investigators.	5
Data items	11	List and define all variables for which data were sought and any assumptions and simplifications made.	5–6
Critical appraisal of individual sources of evidence	12	If performed, provide a rationale for conducting a critical appraisal of included sources of evidence; describe the methods used and how this information was used in any data synthesis (if appropriate).	-
Synthesis of results	13	Describe the methods of handling and summarizing the data that were charted.	5–6
**Results**			
Selection of sources of evidence	14	Give the number of sources of evidence screened, assessed for eligibility, and included in the review, with reasons for exclusions at each stage, ideally using a flow diagram.	6
Characteristics of sources of evidence	15	For each source of evidence, present characteristics for which data were charted and provide the citations.	7–12
Critical appraisal of sources of evidence	16	If performed, present data on critical appraisal of included sources of evidence (see item 12).	-
Results of individual sources of evidence	17	For each included source of evidence, present the relevant data that were charted that relate to the review questions and objectives.	7–12
Synthesis of results	18	Summarize and/or present the charting results as they relate to the review questions and objectives.	6–12
**Discussion**			
Summary of evidence	19	Summarize the main results (including an overview of concepts, themes, and types of evidence available), link to the review questions and objectives, and consider the relevance to key groups.	12–17
Limitations	20	Discuss the limitations of the scoping review process.	16–17
Conclusions	21	Provide a general interpretation of the results with respect to the review questions and objectives, as well as potential implications and/or next steps.	18
**Funding**			
Funding	22	Describe sources of funding for the included sources of evidence, as well as sources of funding for the scoping review. Describe the role of the funders of the scoping review.	18

## Appendix B

**Table A2:** Main Descriptive Characteristics and Results from the Included Studies.

Service Perspective	Reference, Year, and Country	Objectives	Results	Type of Industry	Stage
Cooking	Anami, Burkpalli (2009) [21] India	To propose a methodology for the identification and classification of boiled food grains based on the level of boiling using two color models, HSV and L*a*b*, in the Indian context.	It proved efficient in identifying the cooking of grains, which can assist in quality control and the development of equipment for the automation of industrial kitchens.	4.0	Test
	Junge et al. (2020) [22] United Kingdom	To introduce the concept of food quality optimization and its challenges with an automated omelet cooking robotic system.	It demonstrated the potential for optimized preparation and the ability to personalize preparations for a group of individuals with distinct dietary preferences.	5.0	Test
	Ronzoni et al. (2021) [23] Italy	To provide a novel support–design framework for the cooperative robot system in labor-intensive manufacturing processes to aid layout and task scheduling design.	The proposed layout of a collaborative cobot for the end-of-line that is accessible, ergonomically convenient, and safe for workers. The test bench confirmed that it is a facilitating tool for the integration of human–robot technologies in current manufacturing systems under budgetary and workforce constraints. It improved performance, safety, and worker satisfaction.	5.0	Test
Customer Services	Acosta et al. (2006) [24] Spain	To present a highly specialized autonomous robot.	The tested robot successfully classified the various types of utensils, proving efficient in collecting utensils from tables.	4.0	Test
	Tan et al. (2011) [25] China	To integrate radio frequency identification (RFID), wireless local area network, database technologies, and a menu recommender to develop an intelligent e-restaurant for customer-centric service.	It allowed waiters to identify customers by RFID and recommend personalized menus with good practical potential for offering a customer-centric service.	5.0	Test
	Cheong et al. (2016) [26] Singapore	To develop a prototype of a mecanum-wheeled based mobile waiter robot for trial runs in a casual dining food outlet.	The robot performed well in serving customers, but improvements were suggested to enhance its agility and localization accuracy.	4.0	Test
	Li et al. (2018) [27] China	To conduct an exploratory study on an intelligent food choice method that recommends dishes by predicting an individual’s dietary preference, including ingredients, types of spices, price, etc.	AI using the MAR MTF (multi-attribute relation matrix tri-factorization—an algorithm that combines attributes), combining data on ingredients, seasonings, and prices, demonstrated significantly superior performance compared to traditional models for accuracy in predicting consumer food preferences, recommendations, and service personalization.	5.0	Test
	Roanes-Lozano et al. (2019) [28] Spain	To develop a prototype of a functional approach to personalized menu generation using set operations.	It showed speed in generating a menu instantly according to the customer’s dietary characteristics and the availability of ingredients in the restaurant.	5.0	Test
	Tallam, Joseph (2019) [29] India	To focus on the movement of a robot in identified locations based on the Bluetooth module.	It demonstrated operational efficiency by successfully connecting to mobile devices, recognizing table numbers, and delivering orders accurately; the use of infrared sensors aided navigation and obstacle prevention, ensuring efficient movement and reducing service time.	4.0	Test
	Chen et al. (2022) [30] China	To introduce the development of a service robot that is designed for food service in fast food restaurants with the innovative improvement of mapping, localization, and navigation.	Compared to well-trained waiters, the robots took longer to serve (1 min for waiters vs. 2 to 3 min for robots). Delivery interruptions occurred in 18% of attempts due to obstacles in the planned path; an additional 14% due to network interruption failures; and 9% due to dirt detection errors in their scanner sensor. Considering these issues along with the high acquisition and maintenance costs, there was a refusal to use robots by the majority of services.	5.0	Implemented
	Huang et al. (2022) [31] China	To propose a dish scheduling model for traditional Chinese restaurants based on hybrid multiple criteria decision-making (MCDM) algorithms and a double-layer queuing structure (DLQS).	The system was able to analyze and establish the relationship between classification rules and the characteristics of customer orders to choose the ideal schedule for the production of preparations and the delivery of dishes to customers.	5.0	Test
	Shimmura et al. (2023) [32] Japan	To introduce service robots to a restaurant company to reduce work hours and improve service quality.	The robot led to a reduction in working hours, increased productivity, and improved employee motivation and collaboration. There was no significant difference in overall sales, but there was a significant difference in sales per person/hour.	5.0	Implemented
	Sultana et al. (2024) [33] India	To propose a quick-witted, intelligent order-handling system utilizing the Internet of Things (IoT) to enhance the overall dining experience.	It was observed that an increase in customer return rates, greater order accuracy, and improved resource management led to increased profitability and operational efficiency and enhanced service and customer loyalty.	5.0	Implemented
Management	Blasi (2018) [34] Jordan	To present a schedule for the food industry using a fuzzy-based control system and focus on determining the number of ovens and workers needed for a certain job in the food manufacturing system, especially the pizza production industry.	It reduced customer waiting times and operational costs, improving production efficiency and preventing resource waste; it enhanced the quality of customer service by ensuring on-time deliveries in the desired quantity.	4.0	Test
	Sun et al. (2018) [35] China	To propose a loss prevention method for the catering enterprises based on a machine learning algorithm.	It efficiently identified normal and exceptional service events that could lead to financial losses impacting profitability. The continuous analysis of operational data allowed for adjustments in service practices, improved employee and billing management, and provided greater profitability.	4.0	Implemented
	Tufano et al. (2018) [36] Italy	To propose an original computer-based multidisciplinary decision support tool for the design and configuration of a CEKI.	The system aided multiprofessional decision-making simultaneously with the evaluation of performance indicators and scenario simulation analyses. It achieved a 3.7% increase in net profit and an energy saving of 12.9%, along with a reduction in customer waiting time and the total cost of production.	5.0	Test
	Tom, Anaraud (2021) [37] USA	To present a Menu Management Decision Support Model (MMDSM) that applies the Fuzzy Multicriteria Decision-Making (FMCDM) approach to deal with the inherent imprecision in input using qualitative linguistic input values and obtain reliable outputs with increased decision options.	The AI with the tested model successfully produced reliable recommendations for a restaurant manager to make strategic decisions based on automated menu analysis.	5.0	Test
	Gomez-Talal et al. (2024) [38] Spain	To address the global food waste problem in restaurants by analyzing customer sales information provided by restaurant tickets to gain valuable insights into directing sales of perishable products and optimizing product purchases according to customer demand.	Big Data (BD) and ML technologies have aided in analyzing restaurant customer orders to target sales of perishable goods and optimize product purchases according to demand, thereby benefiting restaurant procurement management.	5.0	Test
	Groene, Zakharov (2024) [39] Germany	To propose an approach to work with F&B owners in creating and introducing machine learning (ML)-based sales forecasting tailored to the unique local aspects of the business.	The automation of sales forecasting by the AI model has demonstrated superiority over other forecasting models and has freed managers from repetitive tasks.	4.0	Test
	Hao et al. (2024) [40] USA	To investigate the integration of Blockchain technology into the food supply chain within the restaurant industry.	It indicated that the adoption of Blockchain significantly enhances traceability and trust in the food supply chain, which can increase customer satisfaction through perceived improvements in food safety, quality, and freshness. It was observed that the effects vary depending on the type of restaurant (casual or fine dining) and its location (tourist or residential areas).	5.0	Test
Quality control	Bhatia (2020) [41] India	To present an IoT-inspired framework to analyze restaurants and food hubs from a food quality perspective.	The correlation analysis conducted identified the best model and demonstrated that the use of a system with game theory and IoT technology can be a viable approach to improve food quality control in restaurants, providing real-time evaluation and statistically reliable results.	5.0	Implemented
	Bhatia, Manocha (2022) [42] India	To propose a novel notion of smart restaurants for assessing food quality using game theory.	The proposed use of AI, Fog Computing, and Cloud Computing technologies was effective in evaluating food quality in real-time, outperforming other decision-making techniques in terms of temporal and classification effectiveness, statistical efficiency, and reliability.	5.0	Test
Sustainability	Aytaç, Korçak (2021) [43] Tukey	To propose an edge-oriented IoT architecture for Quick Service Restaurants.	The system was efficient in detecting anomalies and discrepancies in the disposal of food waste, as well as in estimating production in advance. Furthermore, the system was capable of making decisions based on the collected data, which resulted in a reduction in food waste.	5.0	Test
	Eriksson et al. (2021) [44] Sweden	To evaluate the potential of a forecasting model to predict guest attendance during the start and throughout the pandemic.	Conventional forecasting methods (total number of students or the last number of meals served or the average number of meals served) were simple to use and presented the best results in 7 dining halls. However, the use of AI with a neural network model demonstrated the best performance in 11 of the 21 dining halls studied, proving to be a more sustainable and resilient option for the public food service sector.	5.0	Test
	Faezirad et al. (2021) [45] Iran	To propose a new model based on machine learning to reduce the food waste generated at major universities that provide food subsidies.	The model collected student reservation data, the attendance or no-show rate at the dining hall, and meal times. The analysis of this data enabled better adjustment of meal production and a reduction in total cost, potentially reducing food waste (not measured).	5.0	Test
	Gull et al. (2021) [46] Pakistan	To introduce a new approach by utilizing some low-cost sensors.	The system presented an accuracy rate of 92.65% for alerts concerning food deterioration signals and for the assessment of food wastage.	5.0	Test
	Malefors et al. (2021) [47] Sweden	To examine conventional forecasting methods (last-value forecasting, moving-average models) and more complex models (prophet model, neural network model), and calculate associated margins for all models.	The comparative evaluation of multiple machine learning (ML) methods revealed that the random forest algorithm yielded more accurate predictions than a simple artificial neural network (ANN). Furthermore, a significant cost reduction was observed with the implementation of ML-based forecasting.	4.0	Test
	Principato et al. (2023) [48] Italy	To demonstrate that by using digital tools to monitor food provisioning and management within canteens, it is possible to achieve a more sustainable food service management within industrial companies and to reduce the overall quantity of food produced and environmental impacts of food preparation and consumption, thus achieving economic benefits.	The utilization of the Winnow Waste Monitor system resulted in a 30% reduction in meal production waste over two years, whereas plate waste exhibited a marginal decrease. The optimal AI model for demand forecasting and data management was identified; furthermore, a significant reduction in estimated greenhouse gas emissions from food production was achieved, from −18.3% in 2018 to −30.7% in 2019.	5.0	Implemented
	Rodrigues et al. (2024) [49] Brazil	To propose four models meant to help food catering services predict food demand accurately and thus avoid overproducing or underproducing.	The demand forecasting models based on machine learning exhibited greater efficacy in mitigating food waste compared to traditional forecasting models (referencing the previous day’s figures or empirical prediction); the former demonstrated the capacity to accurately predict next-day demand, thereby contributing to the reduction in waste and costs.	4.0	Test
	Sigala et al. (2025) [50] Germany	To assess the effectiveness of an intervention employing an AI-based, fully automatic waste-tracking system for food waste reduction in HORECA establishments.	The AI-driven food waste analysis system assessed the total waste across different sites, identifying the restaurant with the highest waste at 151.9 g per meal. A significant portion of this waste, ranging from 45% to 73%, was considered avoidable, primarily due to overproduction and food left on consumers’ plates. Vegetables and prepared foods made up the largest volume of waste. With the exception of one hotel that saw an increase in waste, the intervention effectively reduced food waste by 23% to 51%. This reduction was especially notable in areas of food preparation and overproduction, resulting in a decrease in food waste costs per meal of up to 39%. The system proved to be suitable for quantifying food waste related to operational practices and demand forecasting, while also raising staff awareness about waste and enabling the implementation of corrective actions.	5.0	Test
	Turker (2025) [51] Turkey	To tackle food waste by analyzing daily campus data with machine learning and revealing strategic insights related to food variety and sustainability.	The analyses identified preferred food items, predicted meal quantities, and highlighted preferences across diverse consumption areas, utilizing high-performing models.	5.0	Implemented

Source: study data.
